# Single-molecule study of the dynamics of the molecular chaperone Hsp70 during the functional cycle

**DOI:** 10.1042/BST20230831

**Published:** 2025-04-23

**Authors:** Huimin Hu, Ming Yang, Sarah Perrett, Si Wu

**Affiliations:** 1 Key Laboratory of Biomacromolecules (CAS), National Laboratory of Biomacromolecules, CAS Center for Excellence in Biomacromolecules, Institute of Biophysics, Chinese Academy of Sciences, Beijing 100101, China; 2 University of the Chinese Academy of Sciences, Beijing 100049, China; 3 Yusuf Hamied Department of Chemistry, University of Cambridge, Cambridge, CB2 1EW, United Kingdom

**Keywords:** conformational dynamics, heat shock proteins, molecular chaperones, protein folding, single-molecule force spectroscopy, single-molecule FRET

## Abstract

The 70-kDa heat shock protein, Hsp70, is a key chaperone involved in cellular protein homeostasis. The structure of the Hsp70 protein family is highly conserved, including a nucleotide-binding domain (NBD) and a substrate-binding domain (SBD). ATP binding and hydrolysis in the NBD of Hsp70 regulates the binding and release of substrates in the SBD via interdomain allosteric communication. Growing evidence shows that the conformational dynamics of Hsp70 are crucial for its function, which are difficult to probe by traditional bulk-based methods. Single-molecule techniques are emerging as powerful tools to explore the dynamics of proteins that are obscured in bulk measurements. In this review, we summarize recent progress in the study of the molecular dynamics of Hsp70 and its interactions with cochaperones and substrates using single-molecule fluorescence spectroscopy and single-molecule force spectroscopy. We discuss how the application of single-molecule techniques facilitates a deeper understanding of the mechanistic details of the chaperone functions of Hsp70.

## Introduction

**Figure 1:**
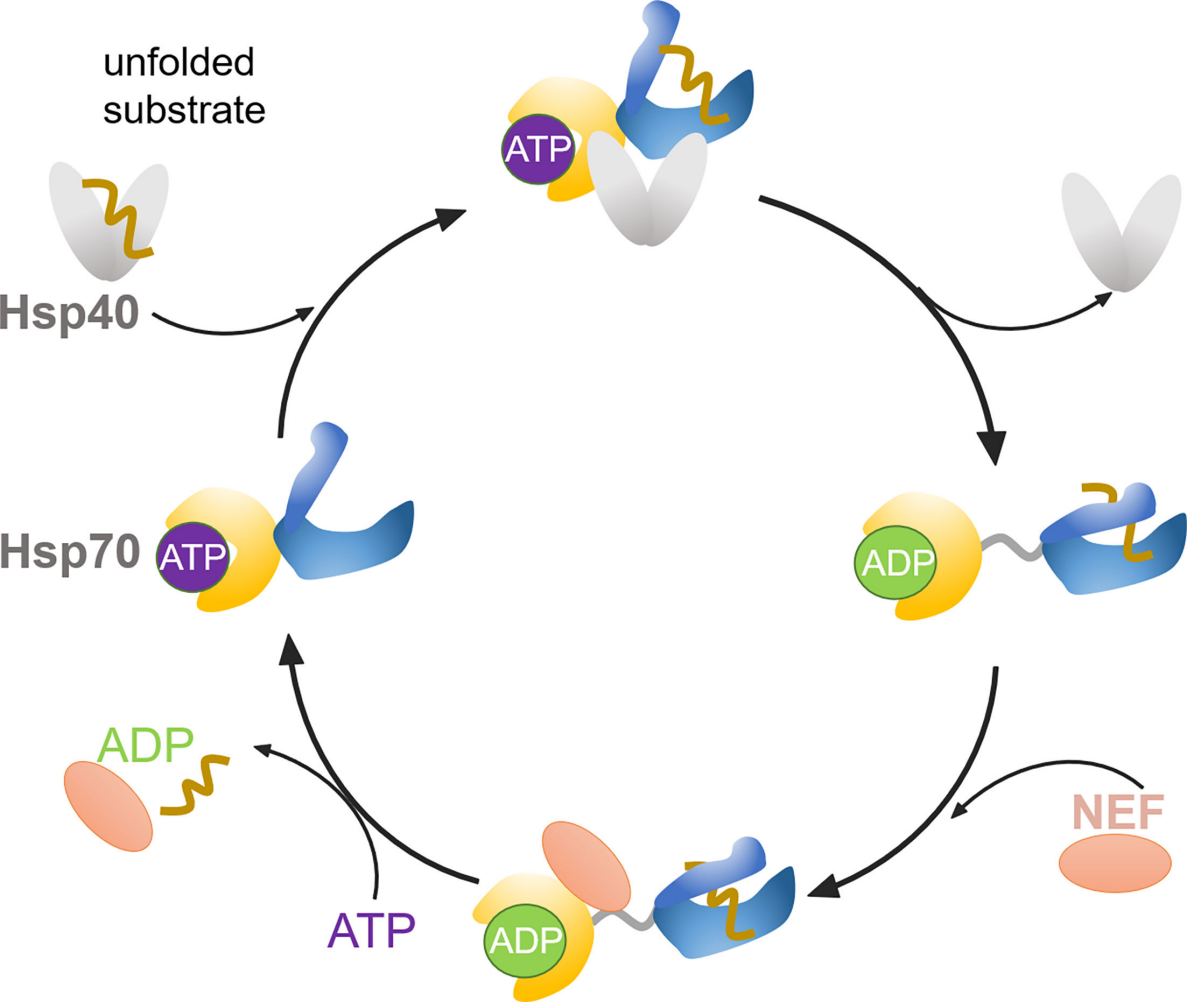
Schematic figure illustrating the conformational cycle of Hsp70. Single-molecule study of the conformational cycle of Hsp70 reveals the importance of dynamics for its function. Hsp40 (gray) interacts with ATP-bound Hsp70 (NBD in yellow and SBD in blue) and transfers the unfolded substrate protein to the open SBD of ATP-Hsp70. The interaction of Hsp40 with ATP-Hsp70 promotes ATP hydrolysis, causing transition to the ADP-bound state. This transition results in the closure of the SBD of Hsp70 and high binding affinity of substrate. NEF (orange) accelerates the dissociation of ADP and rebinding of ATP, enabling substrate release and initiating a new cycle. Hsp40, 40-kDa heat shock protein; Hsp70, 70-kDa heat shock protein; NBD, nucleotide-binding domain; NEF, nucleotide exchange factor; SBD, substrate-binding domain.

The 70-kDa heat shock proteins (Hsp70s) form the central hub of the proteostasis network, which is involved in a number of cellular processes, including assisting the folding of nascent peptide chains, preventing aggregation of misfolded and unfolded proteins, and disaggregating protein aggregates and degradation of misfolded proteins [1,2]. Due to its involvement in diverse cellular pathways, Hsp70 is currently considered a potential therapeutic target for both cancer and neurodegenerative diseases [3,4]. Hsp70 family proteins are highly conserved from prokaryotes to eukaryotes in both sequence and structure. They are composed of an N-terminal nucleotide-binding domain (NBD) and a C-terminal substrate-binding domain (SBD), which are tethered by a conserved hydrophobic linker. The NBD contains two lobes that form a cleft for nucleotide binding. The SBD includes a substrate-binding cavity formed by a β-sandwich subdomain (SBDβ) and an α-helical bundle lid (SBDα) [5]. ATP binding and hydrolysis in the NBD regulates the substrate-binding affinity of the SBD via interdomain allosteric communication [6,7]. In the ADP-bound and nucleotide-free states, the NBD and SBD of Hsp70 are detached and the SBDα is closed over the SBDβ, leading to slow binding/release kinetics and high binding affinity for substrates [8]. When ATP is bound, the SBD is open with the SBDβ and SBDα docked to different parts of the NBD [9,10], resulting in fast on/off rates and low binding affinity for substrates (Figure 1). Hsp70s usually co-operate with a network of cochaperones, including Hsp40s and nucleotide exchange factors (NEFs) [11]. Hsp40 interacts with Hsp70 to promote transfer and binding of substrate to Hsp70 and to stimulate the ATPase activity of Hsp70 [12]. Upon ATP hydrolysis, NEFs accelerate the release of ADP and substrates and rebinding of ATP to the NBD of Hsp70, and thus, they reset Hsp70 ready for a new cycle [13] (Figure 1). The synergism of Hsp40/NEF allows Hsp70 to cycle efficiently between high- and low-affinity states for substrate binding and promotes its ATP-dependent protein folding, unfolding, and disaggregation activities [14,15].

Although the atomic structures of Hsp70 alone as well as in complex with cochaperones have been resolved by structural biology methods [8–10,16–18], these studies provide only static or snapshot representations of Hsp70. The dynamic nature of Hsp70, which is crucial for its chaperone activity, has only recently started to garner attention. The conformational changes of Hsp70 that are regulated by different nucleotides, substrates, and co-chaperones, as well as by the dynamic processes involved in Hsp70-assisted substrate folding and unfolding, are crucial aspects for understanding the chaperone function of Hsp70. During the past decade, significant progress has been made in unraveling the dynamic mechanism of Hsp70 through the development and application of advanced biophysical techniques, such as NMR [19–23], electron paramagnetic resonance (EPR) [18,24], double electron-electron resonance [25], mass spectroscopy [25,26], and molecular dynamics simulations [27–29]. However, ensemble-based methods still have limitations in exploring the conformational dynamics of chaperones and transient chaperone–substrate interactions.

The emergence of single-molecule techniques, including single-molecule fluorescence spectroscopy and single-molecule force spectroscopy, provides powerful tools for investigating protein dynamics. These techniques allow the observation and analysis of the behavior of individual protein molecules in real time, offering a level of detail that is unattainable through traditional ensemble methods [30,31]. By applying single-molecule techniques, the dynamic behavior and population of transient intermediates during the functional cycle of chaperones such as Hsp60, Hsp70, and Hsp90 have been observed [32,33], which are otherwise averaged out in bulk measurements. Moreover, investigations at the single-molecule level have shed light on how chaperones interact with clients and guide them toward their native state in various capacities as holdases, foldases, or unfoldases, facilitating our understanding of different chaperone functions [34–37]. In this review, we will particularly focus on recent progress regarding the study of molecular dynamics of Hsp70 and its interactions with cochaperones and substrates by single-molecule techniques, aiming to provide mechanistic insights into this intricate chaperone machinery at the single-molecule level.

### Conformational dynamics of Hsp70 studied by single-molecule fluorescence spectroscopy

**Figure 2:**
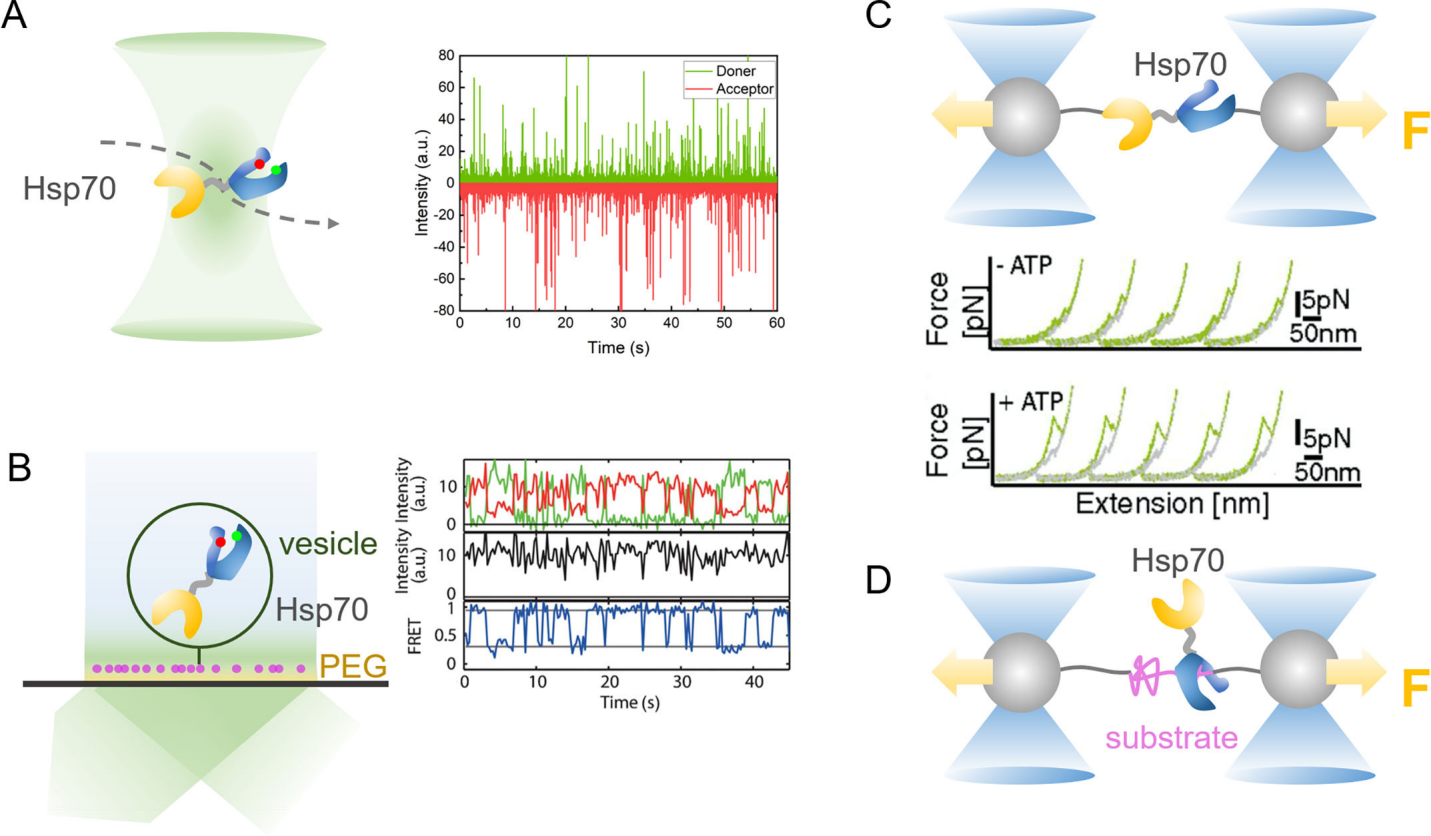
Application of single-molecule techniques to investigate the dynamics of Hsp70. (**A**) Application of confocal single-molecule FRET to detect dual-labeled Hsp70 as it diffuses across the focal volume. The fluorescence bursts from both donor (green) and acceptor (red) channels are recorded, enabling calculation of the FRET efficiency histogram. (**B**) Application of TIRF single-molecule FRET to detect the dual-labeled Hsp70 tethered by surface-immobilized liposomes. Real-time recording of fluorescence intensity traces for both donor (green) and acceptor (red) allows the analysis of conformational dynamics through calculation of the time trace for FRET efficiency (blue). Figure reproduced from ref [42]. (**C**) Application of optical tweezers to study the dynamics of Hsp70 by tethering a single Hsp70 molecule between microbeads on which the pulling force can be exerted. The mechanical properties and folding/unfolding dynamics of Hsp70 can be analyzed based on the consecutive force–extension curves. Figure reproduced from ref [43]. (**D**) Application of optical tweezers to study the chaperone activity of the Hsp70 machinery on the substrate folding/unfolding transitions. FRET, Förster resonance energy transfer; Hsp70, 70-kDa heat shock protein; TIRF, total internal reflection fluorescence.

Single-molecule Förster resonance energy transfer (smFRET) has been applied extensively to investigate the conformational dynamics of proteins, revealing distinct conformational states and the dynamics of their interconversions [30,38]. In smFRET experiments, a pair of residues on the target protein with an appropriate distance and reactivity are selected for site-specific labeling with a donor and an acceptor without interfering with protein function. The most commonly employed approach is to introduce two cysteine residues at specific sites through site-directed mutagenesis, which can subsequently be labeled using maleimide-functionalized fluorophores [39]. In addition, bio-orthogonal labeling strategies via incorporation of unnatural amino acid or peptide tags can be applied when cysteine labeling is not feasible [40,41]. The smFRET measurements are typically performed based on confocal microscopy or total internal reflection fluorescence (TIRF) microscopy. Confocal smFRET is suitable for studying the conformational dynamics of freely diffusing molecules in solution. The fluorescently labeled proteins can be detected with nanosecond time resolution as they pass across the femtoliter focal volume. However, the short diffusional time of molecules in the focal volume (sub-ms to several ms) hinders the analysis of slow dynamics (Figure 2A). In the TIRF setup, fluorescently labeled molecules are either directly immobilized on the surface of the coverslips or encapsulated in lipid vesicles that are immobilized on the coverslips. The molecules are illuminated by the evanescent wave generated at approximately 100 nm above the interface and captured using a camera (Figure 2B) [30]. The TIRF setup allows long-term observation of individual molecules before photobleaching, albeit with a temporal resolution limited to milliseconds by the camera. FRET efficiency (*E*) can be calculated for each molecule based on the signal intensity in both donor and acceptor channels. The change in distance (*r*) between the dye pair is reflected by alterations in FRET efficiency, as described by *E* = 1/[1+(*r*/*R*_0_)^6^]. Consequently, it enables the analysis of protein conformational populations and dynamic transitions.

Confocal smFRET was first applied to investigate the effects of nucleotides on the conformations of the mitochondrial Hsp70 Ssc1 from yeast and of DnaK from *Escherichia coli* [44]. The Hsp70 variants were constructed with fluorophore labeling on NBD/SBDβ and SBDβ/SBDα to monitor NBD-SBD docking/undocking and SBD opening/closing, respectively. The conformations of ATP-bound Ssc1 and DnaK were found to be homogeneous with docked NBD-SBD and open SBD, while the ADP-bound Ssc1 shows heterogeneity with respect to the NBD-SBD interactions and the conformation of the SBD, different from ADP-bound DnaK, which shows completely undocked NBD and SBD even though it populates both closed and partially open SBD [44]. Conformational heterogeneity has also been observed for the endoplasmic reticulum (ER) Hsp70 BiP [45]. The existence of multiple conformations between the NBD and SBD was not only observed for apo and ADP-bound BiP but also for AMPPNP-bound BiP. Similar to DnaK and Ssc1, the SBD of BiP populates closed and open conformations, as well as an intermediate state in the apo and ADP-bound states. The conformations of Hsp70 SBD can be modulated upon binding to substrates of different sizes [45]. The conformational flexibility of the SBD revealed by smFRET is also consistent with the results obtained by EPR study of DnaK [24]. The dynamic fluctuations of the SBD may enable the Hsp70 molecules to accommodate different kinds of substrates ranging from unfolded proteins to folded proteins and large aggregates *in vivo*, allowing it to assist protein holding, folding, or disaggregation.

**Figure 3:**
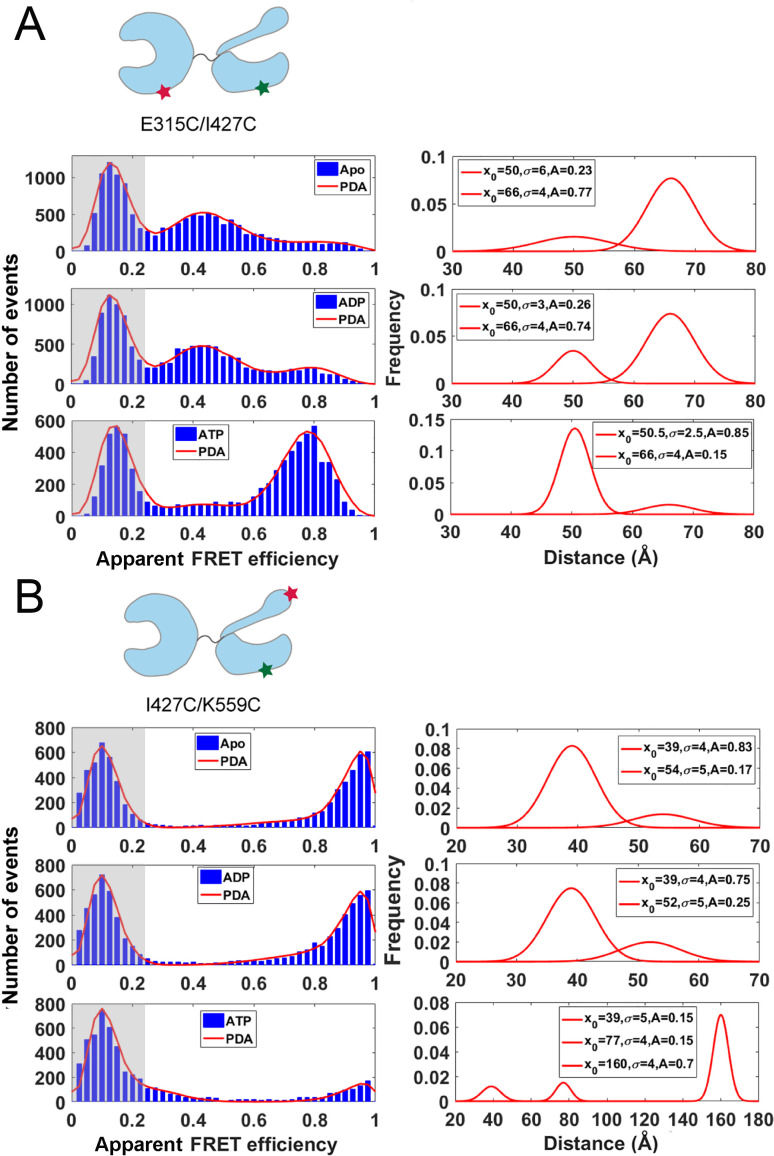
Single-molecule FRET measurements of the conformational populations of human Hsp70 in different nucleotide-bound states. (**A,B**) The smFRET distribution histograms of NBD–SBDβ-labeling variant Cy3/Cy5–Hsp70-E315C/I427C (**A**) and SBDβ–SBDα-labeling variant Cy3/Cy5–Hsp70-I427C/K559C (**B**) in apo, ADP-bound, and ATP-bound states. The low FRET efficiency populations (zero peak) caused by inactive acceptor dye are shaded in gray. The smFRET histograms are fitted by probability distribution analysis (left panels, red lines). The obtained distance distributions between the labeling sites are plotted on the right panels. Figure reproduced from ref [46]. Hsp70, 70-kDa heat shock protein; NBD, nucleotide-binding domain; SBD, substrate-binding domain; smFRET, single-molecule Förster resonance energy transfer.

Although Hsp70 homologs are highly conserved in sequence and structure, a difference in the landscapes of interdomain interactions between *E. coli* DnaK and human Hsp70 has been suggested [20]. Recently, our group applied smFRET to investigate the interdomain conformations of human Hsp70A1 in different nucleotide-bound states [46]. In the apo and ADP-bound state, Hsp70A1 adopts ~75% NBD-SBD docked population and ~25% undocked population, while in the ATP-bound state, it adopts 85% docked and 15% undocked populations (Figure 3A). Meanwhile, the SBD of Hsp70A1 was also found to display both closed and partially open conformations (Figure 3B). Compared with *E. coli* and yeast Hsp70s, human Hsp70 shows higher dynamics in terms of interdomain interactions, which may indicate the increases in complexity of Hsp70 to adapt to more complex physiological environments along the course of evolution.

The effects of cochaperones on the conformational dynamics of Hsp70 have been studied, revealing how these factors promote the functional cycle of Hsp70. The addition of the yeast mitochondrial Hsp40 Mdj1 results in the emergence of the NBD-SBDβ undocked and the SBD closed populations of Ssc1 [44]. Similarly, the interactions of human Hsp40 Hdj1 with Hsp70A1 also increase the proportion of the NBD-SBDβ undocked and the SBD closed conformations [46]. It has been suggested that the dominant NBD-SBDβ docked conformation in ATP-bound Hsp70 is incompatible with ATP hydrolysis [47]. Binding to Hsp40 leads to an ‘ADP-like’ conformation, which probably corresponds to an ATPase-stimulated state, thus promoting the efficiency of the functional cycle of Hsp70. Surprisingly, the ER Hsp40 ERdJ3 only has a minimal effect on the conformations of ATP-bound BiP but induces the ADP-bound BiP to adopt an SBD open, substrate-accepting state [45]. Therefore, the effects of different types of Hsp40s on Hsp70 and how they determine the functional diversity of Hsp70s still need further exploration. As an another important factor in the functional cycle of Hsp70, NEFs have been found to interact with both the NBD and SBD of Hsp70 [16,48,49]. Recent smFRET studies revealed that Bap, an NEF in the ER, can affect the conformations of BiP, especially in the ADP-bound state [50]. Both the unstructured N-terminal domain and the globular C-terminal domain of Bap interact with the SBD lid of BiP, resulting in an SBD open population of ADP-BiP and increased NBD-SBD docked populations. Such conformational changes promote nucleotide release, as well as substrate release from BiP and prepare the BiP for a new ATP-dependent functional cycle [50].

The application of TIRF smFRET allows the direct observation of the conformational dynamics of individual Hsp70 molecules in real time. By encapsulating the dual-labeled Ssc1 in liposomes immobilized on the coverslip, the interdomain dynamic transitions of Ssc1 were monitored for minutes until the photobleaching of the dyes [42]. In the ADP-bound state, by analyzing the dwell times on each state, the transition rates between different conformational states were found to be on the timescale of seconds, with the rate of NBD-SBD docking/undocking faster than SBD opening/closing. Binding to peptide substrate leads to the loss of interdomain dynamics of Ssc1. In contrast, the ATP-bound Ssc1 showed no dynamics between SBDβ and SBDα and occasional undocking of the NBD and SBD [42]. Therefore, TIRF smFRET experiments reveal that the conformational heterogeneity of Hsp70 is due to the dynamic fluctuations between different conformations rather than heterogenous static populations.

While two-color smFRET can only detect a single distance at a time, three-color smFRET allows the simultaneous detection of the coordinated motions of NBD-SBD and SBDβ-SBDα of Hsp70 during the conformational cycle [51]. Using three-color smFRET, the allosteric conformational changes of DnaK, Ssc1, and BiP were studied [52]. The results indicate that the motions of the domains lack coordination and are partially uncoupled, rather than the well-known picture of Hsp70 in which the opening/closing of the SBD correlates with the docking/undocking of the NBD-SBD. Upon the addition of peptide substrate or NEFs, additional intermediate conformations of Hsp70 are adopted that are between the ADP-bound and the ATP-bound states [52]. Overall, the three-color smFRET study reveals not only the existence of conformational heterogeneity but also partial uncoupling of the motions of the NBD and SBD of Hsp70, suggesting more complicated dynamics in the allostery of Hsp70 than previously thought.

### Conformational dynamics of Hsp70 studied by single-molecule force spectroscopy

Single-molecule force spectroscopy, including optical tweezers (OT), magnetic tweezers (MT), and atomic force microscopy (AFM), has proven to be a sensitive tool for exploring the structural dynamics and functions of biomacromolecules [32,53]. In such experiments, individual proteins of interest are immobilized on force probes (cantilever tip for AFM, dielectric beads for OT, and paramagnetic beads for MT) that are actuated by distinct mechanisms, enabling the application of mechanical forces ranging from 10^−8^ to 10^−14^ N to the proteins [31]. In OT and MT experiments, double-strand DNA handles are commonly used as spacers to attach a specific site of the target protein through cysteine–maleimide reaction at one end, with a link to microbeads via biotin–streptavidin or digoxigenin/antibody interactions at the other end. The change of the measured distance upon the application of mechanical force corresponds to alterations in the end-to-end distance of the tethered proteins during their folding and unfolding (Figure 2C and D), thereby providing insights into the protein properties and intermolecular interactions. Beyond detecting equilibrium fluctuations between different conformational states, single-molecule force spectroscopy enables precise manipulation of proteins, thereby providing insights into the mechanical aspects and energetic landscape associated with protein folding/unfolding processes, as well as elucidating the conformational transition pathways at the single-molecule level.

The effects of nucleotide binding on the mechanical stability of the NBD of DnaK were studied using OT [54]. In the apo state, the force–extension curves show that the unfolding process of NBD exhibits triphasic behavior, which corresponds to the unfolding of the C-terminal helix accompanied by the separation of the two lobes of the NBD, subsequent unfolding of lobe II, and finally unfolding of lobe I. Upon nucleotide binding, the C-terminal helix does not significantly contribute to the inter-lobe stability as observed in the apo state. Interestingly, nucleotide binding greatly enhances the stability of lobe II and reverses the mechanical hierarchy between the two lobes [54]. Although the unfolding pathway of NBD differs in detail with or without nucleotide, the overall unfolding forces remain similar regardless of the nucleotide-bound state when the force is applied at the termini. However, when the force is applied to both sides of the nucleotide-binding cleft, the discrimination in unfolding force can be observed, showing higher values in the presence of nucleotide [55]. The unfolding of the NBD exhibits both co-operative and non-co-operative pathways, partition between which can be modulated by mutation or the presence of NEF [55]. The refolding process of the NBD of DnaK was also investigated by OT experiments. By continuously stretching and relaxing single-tethered NBD molecules, two refolding intermediates (RF1 and RF2) were identified, corresponding to lobe II’s partial and complete folding. Further analysis of the equilibrium fluctuations between folded and unfolded conformations provided insights into the kinetics along the folding pathway, revealing that lobe II serves as a stable folding nucleus that is essential for binding of nucleotides and for proper folding of the NBD to the native state [43].

In contrast to the NBD, which unfolds co-operatively as its two lobes are held tightly by the C-terminal helix, the SBD comprises two relatively independent subdomains (SBDβ and SBDα), resulting in bifurcating unfolding pathways that initiate from unfolding of either the SBDβ or SBDα. When pulling the N- and C-termini of the SBD, rapid conformational fluctuations were observed, which were found to be caused by the expansion of the C-terminal strands of the SBDβ and the disruption of the inter-subdomain interface [56]. These force-induced changes may also be implicated in the ATP-induced allosteric changes of full-length Hsp70. A recent study investigated the mechanical properties of the two-domain DnaK (1–552ye) variant using OT [57]. In both apo and ADP-bound states, the NBD and SBD are mechanically stable and exhibit the properties of undocked NBD-SBD and closed SBD. In the ATP-bound state, the SBD became destabilized due to allosteric interdomain docking and unfolding of the SBDα. By monitoring the folding state of the SBD, the real-time observations revealed a strict coupling between SBD closure and ATP hydrolysis during the ATP cycle of DnaK [57]. However, the variant used in this study was reported to possess an unstable SBDα [23], thus rendering it inadequate for representing the mechanical behavior of WT Hsp70. Furthermore, the difference in intradomain and interdomain mechanics among different Hsp70 homologs also needs further exploration.

### Chaperone activity of the Hsp70 machinery studied at the single-molecule level

The primary role of Hsp70 is to assist protein folding and prevent misfolding. In co-operation with Hsp40 and NEF, Hsp70 utilizes the energy of ATP to shift the folding equilibrium of substrates toward the native state [58]. However, how the folding landscape of the substrate is efficiently modulated during the dynamic functional cycle of Hsp70 remains an ongoing area of investigation. Single-molecule techniques have been employed to explore the mechanisms underlying foldase and unfoldase activities of the Hsp70 machinery by directly monitoring the substrate folding states and dynamics. Using confocal smFRET, the influence of DnaK/DnaJ/GrpE (DnaKJE) on the conformations and folding states of the dual-color-labeled multidomain protein firefly luciferase (Fluc) was studied [36]. While the C-domain of Fluc can spontaneously fold to its native structure, the N-domain of Fluc showed a slow folding rate and high-FRET misfolded populations with compact conformations, consistent with the findings from OT experiments [59]. In the presence of DnaK/DnaJ, unfolded or misfolded N-domain was stabilized in an expanded state exhibiting low-FRET populations resembling the denatured state. Subsequent addition of the NEF GrpE resulted in the refolding of Fluc from the expanded state to the native state. This study shows that the Hsp70 machinery unfolds kinetically trapped misfolded substrates and allows their refolding into native structures [36]. Subsequently, the real-time conformational changes of surface-immobilized Fluc during DnaK-assisted folding were observed by TIRF smFRET [35]. By kinetic analysis of the transitions between different conformations along the FRET trajectories, it was revealed that DnaJ selectively binds to and stabilizes the expanded conformation of Fluc, while DnaK, recruited by DnaJ, unfolds the compact misfolded conformations of the substrate by an entropic pulling mechanism [60]. Interestingly, optimal partitioning between the correct folding pathways to the native state versus spontaneous misfolding occurs at intermediate concentrations of GrpE during substrate refolding. Once the released substrate is misfolded, multiple cycles of chaperone binding and release are required for refolding to the native state [35]. For another model substrate, rhodanese, smFRET experiments show similar observations where DnaK in collaboration with DnaJ could convert the denatured rhodanese from a compact misfolded state to expanded conformational ensembles in an ATP-dependent manner. However, the addition of GrpE only accelerates the disappearance of expanded conformations without refolding into the native structure [61], indicating that certain substrates may require additional downstream chaperones for efficient handling of the substrates transferred from the Hsp70/Hsp40 system to assist their refolding to the native state.

Single-molecule force spectroscopy has also been used to investigate the Hsp70-mediated mechanical unfolding and refolding landscape of substrates as shown in Figure 2D [62]. In such studies, tandem substrate proteins are commonly used and subjected to AFM [63,64], OT [34], or MT [65] for continuous stretching and relaxation cycles in the presence or absence of chaperones. Consistent with the observations from smFRET experiments, single-molecule force experiments reveal that DnaJ preferentially binds to the extended state of the substrate, while DnaK shows affinities for the collapsed state, thereby impeding efficient folding [63]. However, when using a different substrate, namely the I27 domain of the titin protein, it was found that DnaJ interacts with collapsed intermediates rather than the unfolded form [63]. In this case, besides functioning as a holdase, DnaJ also facilitates the folding process by potentially constraining the conformational space of substrate during refolding [64]. The synergy of DnaK with DnaJ and GrpE enhances substrate refolding through distinct mechanisms. Specifically, when ubiquitin is employed as a model substrate that can spontaneously refold, the DnaKJE system accelerates the folding kinetics while the folding efficiency remains unchanged [63]. When the aggregation-prone I27 serves as a model substrate, the combination of DnaK and DnaJ reduces the misfolded population and stabilizes the unfolded state, thereby promoting population toward the correctly folded state and significantly enhancing the refolding efficiency after addition of GrpE [63,64]. In order to explore the functional roles of structural elements in the SBD of DnaK in assisting substrate folding, DnaK variants with a truncated lid, DnaK(2-538), or mutation in the binding groove, DnaK(V436F), were employed in substrate stretch–relax cycles. The results demonstrate that the SBDβ groove plays a crucial role in interacting with unfolded substrate, while the SBDα is critical for stabilizing partially folded conformations of substrate [34]. The holdase and unfoldase functions of Hsp70 are not only crucial for biasing the folding equilibrium of substrates toward the correct pathway but also indispensable for regulating the activities of transcription factors [66–68]. In a recent study, the unfoldase activity of Hsp70/Hsp40 on the ligand-binding domain of glucocorticoid receptor (GR-LBD) was investigated using OT [37]. In the absence of chaperones, the N-terminal region of GR-LBD, which acts as a lid for ligand binding, exhibited equilibrium fluctuations between the open and closed states corresponding to holo and apo conformations. Upon the addition of Hsp70 and Hsp40, ATP-dependent stepwise unfolding of GR-LBD occurred, while its refolding process was inhibited. Importantly, it was observed that the J domain of Hsp40 alone could also promote the unfoldase activity of Hsp70, indicating that the stepwise unfolding of GR-LBD was due to the binding of multiple Hsp70 molecules, while Hsp40 primarily stimulated ATP hydrolysis by Hsp70 [37].

To date, the majority of our understanding regarding Hsp70 is derived from studies on the prokaryotic Hsp70 DnaK as a model system. A comprehensive understanding of the differences in conformational dynamics among Hsp70 homologs and how these distinctions relate to their specific physiological functions needs to be further investigated. Additionally, the eukaryotic Hsp70 protein undergoes a diverse array of post-translational modifications (PTMs) in the cell. Further investigation is needed to elucidate how these PTMs affect the conformational dynamics of Hsp70, as well as its interactions with client proteins. Meanwhile, there are also some technical limitations of single-molecule detection that need to be improved. For instance, single-molecule techniques primarily measure long-range distance changes associated with domain motion/folding/unfolding, with limited ability to observe local changes. Techniques sensitive to subtle local changes such as proximity-induced fluorescence quenching provide a complementary strategy to gain insight into the initial rapid stage of structural rearrangement in Hsp70 [69]. Furthermore, in single-molecule experiments, the choice of labeling sites is limited to those that do not interfere with molecular functions, thereby restricting the range of sites that can be probed. Therefore, integrating single-molecule detection with complementary biophysical approaches such as NMR will be necessary to unravel the working mechanism of the complicated Hsp70 machinery in detail.

Given the high relevance of Hsp70 in cancer and age-related neurodegenerative diseases, a number of small molecule inhibitors have been developed to target Hsp70 and Hsp70–cochaperone interactions [70]. The dynamic mechanisms of Hsp70 revealed by single-molecule studies will provide valuable insights into its allosteric regulation, thus facilitating the development of allosteric modulators that may exert more precise control over Hsp70 compared with traditional active site inhibitors. Additionally, discerning the differences in dynamic nature among Hsp70 homologs through single-molecule techniques will aid in designing selective inhibitors, specifically targeting distinct Hsp70 isoforms.

**Perspectives**

- The application of single-molecule techniques has provided deep insights into the conformational dynamics of the 70-kDa heat shock protein, Hsp70, as well as its interactions with cochaperones and substrates. These new insights facilitate fundamental understanding of the molecular properties and working mechanisms of the Hsp70 machinery.
- Based on these achievements, further in-depth investigations can be conducted to elucidate how the conformational dynamics of Hsp70 are intricately adapted for substrate recognition and selection, as well as the substrate transfer between Hsp70 and other pivotal chaperones involved in proteostasis. This will significantly advance our knowledge of the mechanistic aspects underlying Hsp70 functions.
- In future studies, the integration of single-molecule detection with other complementary biophysical approaches will offer multidimensional insights into the dynamics of Hsp70.

## Abbreviations

AFM: atomic force microscopy
EPR: electron paramagnetic resonance
ER: endoplasmic reticulum
FRET: Förster resonance energy transfer
Hsp40: 40-kDa heat shock protein
Hsp70: 70-kDa heat shock protein
MT: magnetic tweezers
NBD: nucleotide-binding domain
NEF: nucleotide exchange factor
OT: optical tweezers
PTM: post-translational modification
SBD: substrate-binding domain
TIRF: total internal reflection fluorescence
smFRET: single-molecule Förster resonance energy transfer
